# In Marfan Syndrome and Related Diseases, STABILISE Technique Should Be Used with Care: Results from a Volumetric Comparative Study of Endovascular Treatment for Aortic Dissection

**DOI:** 10.3390/jcm12134378

**Published:** 2023-06-29

**Authors:** Ron Azogui, Alizee Porto, Maxime Castelli, Virgile Omnes, Mariangela De Masi, Michel Bartoli, Philippe Piquet, Vlad Gariboldi, Tiffany Busa, Alexis Jacquier, Laurence Bal, Marine Gaudry

**Affiliations:** 1 Timone Aortic Center, Department of Vascular Surgery, APHM, Timone Hospital, 13005 Marseille, France; ron.azogui@ap-hm.fr (R.A.); virgile.omnes@ap-hm.fr (V.O.); mariangela.demasi@ap-hm.fr (M.D.M.); michel.bartoli@ap-hm.fr (M.B.); philippe.piquet@ap-hm.fr (P.P.); alexis.jacquier@ap-hm.fr (A.J.); laurence.bal@ap-hm.fr (L.B.); 2 Department of Cardiac Surgery, APHM, Timone Hospital, 13005 Marseille, France; alizee.porto@ap-hm.fr (A.P.); vlad.gariboldi@ap-hm.fr (V.G.); 3 Department of Radiology, APHM, Timone Hospital, 13005 Marseille, France; maxime.castelli@ap-hm.fr; 4 Department of Genetic, APHM, Timone Hospital, 13005 Marseille, France; tiffany.busa@ap-hm.fr; 5 Timone Aortic Center, APHM, Centre de Référence Marfan et Apparentés, 13005 Marseille, France

**Keywords:** TEVAR, STABILISE, Marfan and related syndromes, aortic dissection, comparative study

## Abstract

**Objectives:** Aortic dissection in patients with Marfan and related syndromes (HTAD) is a serious pathology whose treatment by thoracic endovascular repair (TEVAR) is still under debate. The aim of this study was to assess the results of the TEVAR for aortic dissection in patients with HTAD as compared to a young population without HTAD. **Methods:** The study received the proper ethical oversight. We performed an observational exposed (confirmed HTAD) vs. non-exposed (<65 years old) study of TEVAR-treated patients. The preoperative, 1 year, and last available CT scans were analyzed. The thoracic and abdominal aortic diameters, aortic length, and volumes were measured. The entry tears and false lumen (FL) status were assessed. The demographic, clinical, and anatomic data were collected during the follow-up. **Results:** Between 2011 and 2021, 17 patients were included in the HTAD group and 22 in the non-HTAD group. At 1 year, the whole aortic volume increased by +21.2% in the HTAD group and by +0.2% the non-HTAD groups, *p* = 0.005. An increase in the whole aortic volume > 10% was observed in ten cases (58.8%) in the HTAD group and in five cases (22.7%) in the non-HTAD group (*p* = 0.022). FL thrombosis was achieved in nine cases (52.9%) in the HTAD group vs. twenty (90.9%) cases in the non-HTAD group (*p* < 0.01). The risk factors for unfavorable anatomical evolution were male gender and the STABILISE technique. With a linear model, we observed a significantly different aortic volume evolution between the two groups (*p* < 0.01) with the STABILISE technique; this statistical difference was not found in the TEVAR subgroup. In the HTAD patients, there was a significant difference in the total aortic volume evolution progression between the patients treated with the STABILISE technique and the patients treated with TEVAR (+160.1 ± 52.3% vs. +47 ± 22.5%, *p* < 0.01 and +189.5 ± 92.5% vs. +58.6 ± 34.8%, *p* < 0.01 at 1 year and at the end of follow-up, respectively). **Conclusions:** TEVAR in the HTAD patients seemed to be associated with poorer anatomical outcomes at 1 year. This result was strongly related to the STABILISE technique which should be considered with care in these specific patients.

## 1. Introduction

Marfan syndrome (MFS) is an autosomal dominant connective tissue disease caused by a mutation in the fibrillin-1 gene on chromosome 15, which involves the cardiovascular, ocular, and musculoskeletal systems [1]. This disease leads to early arterial wall degeneration causing asymptomatic aortic dilation. There are related heritable syndromes sharing similar risks with a different genetic support.

The management of patient with heritable thoracic aortic diseases (Marfan syndrome and related disorders; HTAD) has improved considerably in recent years, however, the surgical treatment of complicated aortic dissection (AD) in these patients remains controversial. The current recommendations are to practice open descending aortic surgery for complicated AD, and reserve thoracic endovascular aortic repair (TEVAR) in case of aortic rupture based on the higher risk of post-operative complications and of mid- and long-term reoperation for aneurysmal evolution and aortic rupture after TEVAR [2,3]. Such complications are caused either by a progression of the parietal disease or by the appearance of new entry tears [4,5,6]. These current recommendations are based on dated retrospective studies and the progress observed in recent years in endovascular techniques has changed the viewpoint of modern vascular surgery [7,8,9] and currently, many teams offer endovascular treatment as a first-line therapy to these high surgical risk patients [4,10].

However, the long-term results remain controversial with a high rate of type IB endoleak linked to the distal stent graft-induced new entry tear and a persistent patent false lumen after TEVAR.

The stent-assisted balloon-induced intimal disruption and relamination (STABILISE) technique offers a preventive treatment for the TAA with encouraging short-term results, notably in this population [10]. This strategy has the potential to achieve complete repair of the dissected aorta with complete aortic remodeling (thoracoabdominal false lumen (FL) obliteration and relamination with intimal flap reapposition) and could improve the long-term outcomes, reducing the need for future reintervention on distal TAA.

However, in a recent study the authors showed an unusual aneurysmal evolution at the bare-stent level, and especially in HTAD patients [11,12].

The aim of this exposed–non exposed study was to compare the anatomical results of endovascular treatment in AD between the HTAD patients and young patients without a connective tissue disorder (population < 65 y/o). The outcomes were also analyzed regarding the STABILISE or TEVAR-alone techniques.

## 2. Materials and Methods

### 2.1. Population

All the patients included in this study were clearly informed regarding the use of their data for clinical research, and the institutional review board approved the project (approval number PADS21-263).

All patients with an aortic dissection were included in a prospective multidisciplinary follow-up since 2018, ensuring optimal medical treatment (in particular with the systematic introduction of beta-blockers), controlled blood pressure, and discussion on an individual basis in the cases of indication to treat.

Patients treated with TEVAR for residual or type B AD since 2011 were included in this study.

We defined 2 groups of patients treated for complicated AD: the HTAD group and the non-HTAD group (mean age 40 y/o (SD12.0) and 57 y/o (SD8.7), respectively. All patients were referred to the Marfan and related-diseases center of our institution. The molecular diagnosis was performed in the National Department of Genetics using next-generation sequencing in a panel of 25 HTAD-related genes.

The molecular diagnostics for all the patients included in the study were performed by the National Department of Genetics using next-generation sequencing in a panel of 35 HTAD-related genes. A dominant genetic background was excluded in the non-exposed patients by the genetic medical team of the regional specialist center in HTAD. Patients over 65 years old or with a probable tissue disorder without identified pathogenic mutation in the gene panel were excluded from this study.

### 2.2. Perioperative Approach

Hybrid treatment with TEVAR and open supra-aortic debranching in at least two steps remains the first-line therapy at our aortic center when the AD involves the arch, as previously detailed. The decision to extend the proximal landing zone was based on the location of the main new entry tear (on distal anastomosis of the ascending aortic repair, on the arch or in the descending thoracic aorta). In the absence of AD in the aortic arch and when the entry tear was in the descending thoracic aorta, we performed TEVAR on the descending thoracic aorta.

The distal extension of the stent graft was based on the distal extension of the aneurysm. Since 2017, we have added bare-stent deployment in the thoraco-abdominal aorta to induce remodeling of the distal dissected aorta according to the Stent-Assisted Balloon-Induced Intimal Disruption and Relamination in AD Repair (STABILISE) technique. This technique was chosen when the anatomical criteria were favorable.

In elective patients, systematic revascularization of the left subclavian artery was performed to prevent the risk of spinal cord ischemia, and cerebrospinal fluid drainage was performed when there was extensive coverage of the thoracic aorta with a stent graft (>250 mm) in the absence of contraindications.

### 2.3. TEVAR Technique

The stent was deployed using the standard technique as previously described [13,14]. Two different stent grafts were used: C-TAG (WL Gore & Associates Inc., Flagstaff, AZ, USA) and Valiant Navion (Medtronic, Santa Rosa, CA, USA). The choice of the stent was left to the discretion of the surgeon.

### 2.4. STABILISE Technique

We added bare-stent deployment in the distal aorta to induce the remodeling of the distal dissected aorta according to the STABILISE technique, as described by Faure et al. [10].

The distal part of the stent graft should end up in an area where the diameter does not exceed 42 mm over a length of at least 20 mm. When feasible, the proximal stent-graft coverage should usually extend 100–150 mm above the celiac trunk to preserve the intercostal arteries.

#### 2.4.1. Distal Aortic Bare-Stent Deployment

The Zenith dissection endovascular stent (ZDES; Cook Medical, Bloomington, IN, USA) was deployed with a 1-stent body overlap in the stent graft and extension as far as the infrarenal aorta. The 36 mm diameter ZDES was used in the case of a maximum external aortic diameter up to 32 mm, and the 46 mm diameter ZDES was used in the case of a maximum external aortic diameter between 32 and 42 mm.

#### 2.4.2. Management of Visceral Arteries

In cases of visceral or renal branches arising from the false lumen or signs of static malperfusion on preoperative CT scans, we catheterized the targeted vessels before inflating the balloon.

#### 2.4.3. Balloon Dilatation of the Bare Stent

Subsequently, a trilobed balloon catheter (WL Gore & Associates, Inc., Flagstaff, AZ, USA) was inserted. Between the distal end of the stent graft and the proximal end of the ZDES, the balloon expansion was performed to the point of intimal flap disruption, leading to reapposition of the intimal flap on the aortic wall. On completion of the balloon angioplasty, an angiogram was performed to ensure the adequate proximal and distal seal of the false lumen, to assess the false lumen obliteration, and the branch vessel patency in the thoracoabdominal aorta. In cases of visceral or renal artery malperfusion, a bare stent could be deployed.

### 2.5. Epidemiological Data

The demographic recorded data were age, gender, risk factors and medical history such as high blood pressure, tobacco use, diabetes mellitus, dyslipidemia, coronary artery disease, valve disease, heart failure, chronic renal failure, or chronic obstructive pulmonary disease. The operative data analyzed were the history of cardiac surgery, the date, and the type of the AD, the date of the TEVAR, the brand and measures of the endoprosthesis, the landing zone and the length of coverage, the type of treatment performed (TEVAR, TEVAR + STABILISE), and the intraoperative and the follow-up morbi-mortality rate.

### 2.6. Radiological Data

We analyzed the preoperative, 1 year, and the last CT scan available during the follow-up. Image analysis and measurements were performed using three-dimensional imaging software (AW Server, General Electric Company, Boston, MA, USA). We used an automatic segmentation based on voxels. Centerlines were made for the true lumen (TL) and the false lumen (FL) from Valsalva sinus to the coeliac trunk.

### 2.7. Diameter Analysis

Diameter measurements were made at different aortic levels in the perpendicular axis and in the centerline for the TL and FL: 3 at the thoracic level (left subclavian artery, carina, and left inferior pulmonary vein) and 3 at the abdominal level (coeliac trunk, left renal artery, and aortic bifurcation).

### 2.8. Volume Analysis

**Figure 1:**
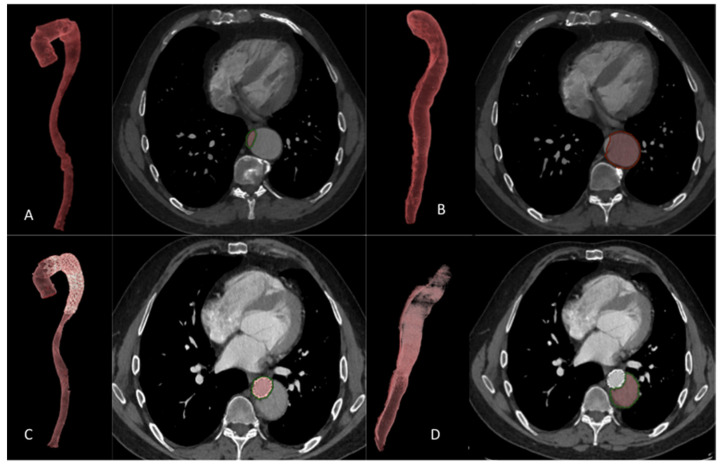
Aortic volume measurement. Aortic volume measurement after true lumen (**A**–**C**) selection and false lumen (**B**–**D**) selection: (**A**,**B**): preoperative CT scan (**C**,**D**): postoperative CT scan.

Volume analysis was performed with semi-automated segmentation which determined the boundaries around the voxels with similar intensity for the TL (Figure 1A–C). For the FL, a manual selection was made on each CT slice and the resulting areas were then multiplied by the length of the midline between the most proximal and distal measurement points (Figure 1B–D). This then allowed the computer software to calculate the volume of the aortic lumen.

On the preoperative CT scan (T0), we measured the aortic volume (TL, FL) from the sinus of the Valsalva to the aortic bifurcation. The total aortic volume was calculated by adding the TL and FL volumes.

On the 1-year (T1) and end-of-follow-up (T2) CT scan, we measured the aortic volume (TL, FL) by separating the aortic segment from the Valsalva sinus to the distal sealing of the covered stent graft and the segment from the distal sealing of the covered stent graft to the aortic bifurcation (thoraco-abdominal aorta below the covered stent graft).

### 2.9. FL Status

The FL patency was assessed as a FL that was enhanced anywhere in the downstream aorta during the arterial- and venous-phase CT, and the FL disappearance was considered complete FL thrombosis.

### 2.10. Entry Tear

The number of entry tears on each CT scan (T0, T1, T2) was reported, the main entry tear was located on the different aortic segments and its diameter was measured. We checked for a new entry tear at the distal landing zone of the stent graft.

### 2.11. Endpoints

Unfavorable anatomical evolution after TEVAR at 1 year (T1) or at the last follow-up (T2) was defined as an increase in the total aortic volume > 10% compared to the preoperative total aortic volume (T0).

Technical success was defined as the exclusion of the lesion or treatment of malperfusion syndrome on perioperative digital subtraction angiography, without perioperative death or surgical conversion.

### 2.12. Statistical Analysis

All analyses were performed using the Statistical Package for Social Sciences software, version 20 (SPSS, IBM Corporation, Armonk, NY, USA). Mean and standard deviation (SD) were used to describe the continuous variables; categorical variables were presented as numbers and frequencies. The HTAD vs. non-HTAD patients were compared. The Mann–Whitney test was used to compare the continuous variables and the categorical variables were compared by χ^2^ or the exact Fisher’s tests. Time-to-event analysis was conducted using the Kaplan–Meier method to estimate the freedom from a second intervention after the TEVAR procedure. The log-rank test was used to compare the HTAD vs. the non-HTAD curve.

The volume of the aorta was compared between the HTAD and the non HTAD patients at the pre-operative, one year and last available CT scans using the nonparametric Mann–Whitney test.

The changes of the aorta volumes were then analyzed using a generalized linear mixed model. The gender, STABILISE technique and patients’ group (HTAD and non-HTAD) were considered as the fixed effects.

All statistical tests were 2-tailed, and *p*-values < 0.05 were considered statistically significant.

## 3. Results

### 3.1. Population (Figure 2)

Between May 2011 and July 2021, seventeen HTAD and twenty-two non-HTAD patients were included in this retrospective study, among them, eight (47.1%) patients in the HTAD group and seven (31.8%) patients in the non-HTAD group were treated with the STABILISE technique according to the standard technique [10]. Two different stent grafts were used: the C-TAG (WL Gore & Associates Inc. Flagstaff, AZ, USA) and the Valiant Navion (Medtronic, Santa Rosa, CA, USA). Sixteen patients were treated in the subacute phase (nine in the HTAD group and seven in the non-HTAD group) and twenty-three in the chronic phase (eight in the HTAD group and fifteen in the non-HTAD group). Indications to treat were malperfusion syndrome (five in the HTAD group and four in the non-HTAD groups), refractory pain (two in the HTAD group), and aneurysmal evolution (ten cases in the HTAD group and eighteen in the non-HTAD groups).

**Figure 2:**
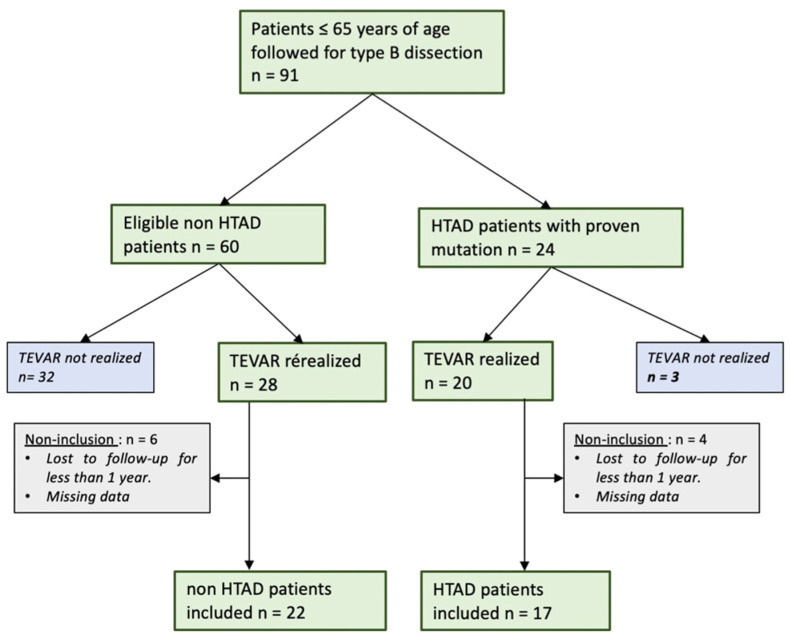
Flowchart.

The mean duration of follow-up was 28.7 ± 20.5 months in the HTAD group and 39.9 ± 31.5 months for the non-HATD group (*p* = 0.362). All patients had radiological follow-up at 1 year and 28 patients had radiological follow-up > 2 years (12 in group 1 and 16 in group 2).

In the HTAD group, the pathogenic mutations were in different genes: *FBN1* (*n* = 10), *SMAD3* (*n* = 2), *COL3A1* (*n* = 1), *TGFB2* (*n* = 1), *TGB3* (*n* = 1), *TGFBR1* (*n* = 1), and *TGFBR2* (*n* = 1).

A comparison of the two group’s characteristics is presented in Table 1 and details of the TEVAR procedures are shown in Table 2.

### 3.2. FL Status

The study of the FL status at the stent graft level found in the HTAD group a patent FL in one case (5.9%), a partially thrombosed in seven cases (41.2%), a complete thrombosed in nine cases (52.9%), and in group two a partially thrombosed in two cases (9.5%) and a complete thrombosed in twenty cases (90.9%) (*p* < 0.01).

A new entry tear at the distal landing zone of the stent graft was observed in seven cases (41.2%) vs. five cases (22.7%) in groups 1 and 2, respectively (*p* = 0.216).

There were two type IA endoleaks vs. zero, in the HTAD and non-HTAD groups, respectively (*p* = 0.18).

The two type IA endoleaks were observed in a patient treated for malperfusion syndrome in residual AD (after type A repair) without primary entry tear exclusion.

There were five type IB endoleaks vs. one, in the HTAD and non-HTAD groups, respectively (*p* = 0.07).

There were three type II endoleaks vs. two, in the HTAD and non-HTAD groups, respectively (*p* = 0.64).

### 3.3. Anatomical Results

####  3.3.1. Diameter Analysis

**Figure 3:**
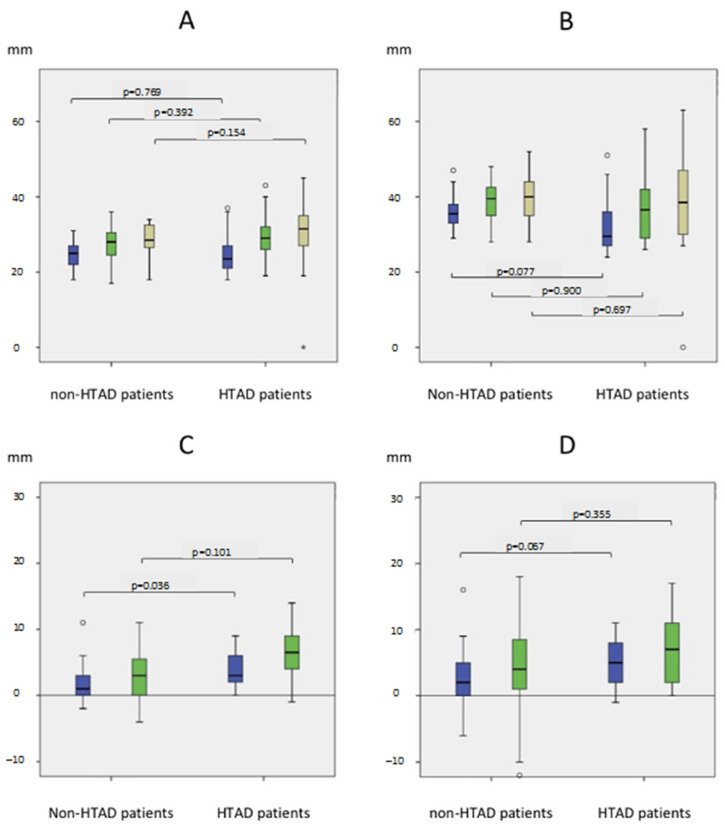
Aortic diameters analysis. Aortic Diameters in mm at (**A**) the aortic bifurcation and (**B**) at the celiac trunk levels (Blue: T0; Green: T1; Khaki: T2). Evolution in mm of the aortic diameters at the aortic bifurcation level (**C**) and at the celiac trunk level (**D**) (Blue: aortic progression at 1 year; Green: aortic progression at the end of follow-up).

The significant changes in diameters are summarized in Figure 3.

At 1 year, there was a significant difference in the diameter changes of the abdominal aorta between É groups: +3.7 mm (±2.8) in the HTAD group vs. +1.3 mm (±4.9) in the non-HTAD group, as well as a significant difference in evolution of the aortic diameters at the celiac trunk level (HTAD group: +5.0 mm (±3.9) vs. non-HTAD group: +1.1 (±8.3); *p* = 0.067).

There was no significant difference in the changes in diameter in the thoracic aorta between the two groups.

#### 3.3.2. Volume Analysis

At T0, the HTAD patients had a lower false lumen volume (264.8 ± 100.4 mL) than the non-HTAD patients (380.2 ± 181.3 mL) (*p* = 0.036), leading to a lower total volume of the aorta (504.5 ± 144.3 mL vs. 652.8 ± 173.5 mL; *p* = 0.005).

At T1 and at T2, no difference in the total aortic volume was observed between the two groups.

##### Results at 1 Year

There was a significantly different change in the total aortic volume between the two groups (+21.2 ± 24.2% vs. +0.2 ± 19.6% in the HTAD and the non-HTAD groups, respectively; *p* < 0.01), with a tendency for the TL volume to increase (HTAD group: +100.2 ± 69.6%; non HTAD group: +62.4 ± 46%; *p* = 0.11). However, no difference in the evolution of the FL was observed between the two groups (HTAD group: −27.7 ± 65.4%; non HTAD group: −35.8 ± 45.4%; *p* = 0.922).

There were significantly more patients with an increase in the aortic volume > 10% in the HTAD group: 58.8% of patients (10/17) compared to 22.7 % (5/22) in the non-HTAD group, *p* = 0.022.

##### Results at the End of the Follow-Up

There was no significant difference in the total aortic volume changes between the HTAD group (+26.4 ± 19.3%) and the non-HTAD group (+9.9 ± 35.9%) (*p* = 0.12). We found the same result for the TL (group one: +124.1 ± 96.1%; group two: +75.7 ± 53.2%; *p* = 0.13). No difference in the FL volume evolution was observed between the two groups (−38.4 ± 40.7% vs. −31.0 ± 51.9% in groups 1 and 2, respectively; *p* = 0.918).

There were significantly more patients with an increase in the aortic volume > 10% (group one: ten patients, 58.8%; group two: five patients, 22.7%; *p* = 0.022).

**Figure 4:**
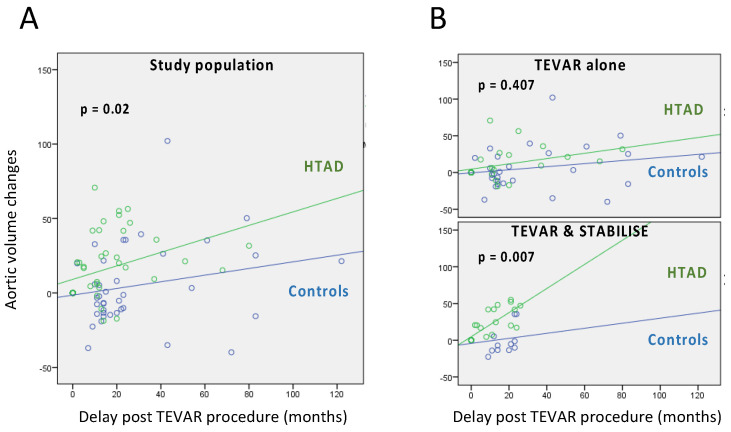
Volume analysis. (**A**) Aortic volume changes in HTAD patients (green curves) vs. controls (blue curves). Changes are expressed in percentage. (**B**) Stratification by surgical technique.

In Figure 4A, with a linear model, we observed a significantly different aortic volume evolution between the two groups (*p* = 0.02).

#### 3.3.3. Subgroup Study: TEVAR/STABILISE: Volume Analysis (Table 3)

Eight (47.1%) patients in group one and seven (31.8%) patients in group two were treated with STABILISE technique.

**Table 3:** Evolution of aortic volumes at 1 year and at last follow-up of the TL, FL and total aorta comparing patients in groups 1 and 2 who had been treated or not with STABILISE.

At 1 Year	True Lumen			False Lumen			Total		
**Group**	non HTAD	HTAD	*p*-value	Non-HTAD	HTAD	*p*-value	Non-HTAD	HTAD	*p*-value
**STABILISE (−) % mean (SD)**	45.7 (±39.7)	47.0 (±22.5)	0.77	−17.5 (±43.3)	6.6 (±73.0)	0.482	1.7 (±20.3)	16.7 (±30.1)	0.263
**STABILISE (+) % mean (SD)**	98.3 (±38.9)	160.1 (±52.3)	**0.029**	−74.9 (±15.2)	−66.3 (±21.9)	0.694	−3.1 (±19.1)	26.2 (±16.4)	**0.009**
***p*** **-value**	**0.011**	**<0.001**		**0.001**	**0.006**		0.447	0.277	
**Last Follow-Up**	**True Lumen**			**False Lumen**			**Total**		
**Group**	Non-HTAD	HTAD	*p*-value	Non-HTAD	HTAD	*p*-value	Non-HTAD	HTAD	*p*-value
**STABILISE (−) % mean (SD)**	71.2 (±59.5)	58.6 (±38.4)	0.967	−17.6 (±52.4)	−13.3 (±39.2)	0.432	12.3 (±40.0)	17.1 (±17.6)	0.773
**STABILISE (+) % mean (SD)**	89.2 (±29.4)	189.5 (±92.5)	0.042	−71.2 (±22.8)	−63.4 (±24.2)	0.648	2.7 (±22.5)	35.7 (±17.2)	**0.042**
***p*** **-value**	0.17	**0.004**		**0.042**	**0.026**		1	0.128	

HTAD: Heritable Thoracic Aortic Disease.

##### Results at 1 Year

In patients who had been treated with the STABILISE technique, the FL volume was stable in both the groups and the TL volume increased by 160% (±52.3) vs. 98.3% (±38.9) in the HTAD- and non-HTAD groups, respectively (*p* = 0.029). The total aortic volume increased by 26.2% (±16.4) in the HTAD group and by 3.1% (±19.1) in the non-HTAD group (*p* < 0.01).

These statistical differences were not found in the TEVAR subgroup.

##### Results at the End of the Follow-Up

In the patients who had been treated with the STABILISE technique, the FL volume was stable in both the groups and the TL volume increased by 189% (±92.5%) vs. 89.2% (±29.4%) in the HTAD- and non-HTAD groups, respectively (*p* = 0.042). The total aortic volume increased by 35.7% (±17.2%) in the HTAD group and by 2.7% (±22.5), in the non-HTAD group (*p* = 0.042).

These statistical differences were not found in the TEVAR subgroup.

In Figure 4B, with a linear model, we observed a significantly different aortic volume evolution between the two groups (*p* < 0.01) with the STABILISE technique.

This statistical difference was not found in the TEVAR subgroup.

### 3.4. Risk Factors for Unfavorable Anatomical Evolution in HTAD Group

In univariate analysis, male gender was significantly associated with an increased risk of aortic progression at 1 year and at the end of follow-up.

At 1 year, there was a significant difference in the total aortic volume evolution progression between the patients treated with the STABILISE technique (+160.1 ± 52.3%) and the patients treated with TEVAR (+47 ± 22.5%), *p* < 0.01.

At the end of the follow-up, there was a significant difference in the total aortic volume evolution progression between the patients treated with the STABILISE technique (+189.5 ± 92.5%) and the patients treated with TEVAR (+58.6 ± 34.8%), *p* < 0.01

There was a significant difference in the increase in the thoraco-abdominal aorta FL volume at the last follow-up between the patients treated with the STABILISE technique (+49.9 ± 43.0%) and the patients treated with TEVAR (−27.0 ± 53.7%) (*p* = 0.03).

### 3.5. Morbi-Mortality

#### 3.5.1. Perioperative Morbidity and Mortality

Technical success was 100% in both groups.

There were no deaths in either group. Perioperative aortic morbidity was 5.9% (1/17) in the HTAD group (one case of retrograde AD one month after TEVAR) and 9.1% (2/22) in the non-HATD group (one renal hemorrhage treated with renal artery embolization and one patient with a medullar hematoma related to cerebrospinal fluid drainage with cauda equina syndrome (loss of bowel and bladder function but no leg paralysis).

#### 3.5.2. Long-Term Morbidity and Mortality

At the end of the follow-up, there was one death (5.9%) in the HTAD group and two (9.1%) in the non-HTAD group.

There was one case (11.8%) of retrograde AD (at 5 years) in the HTAD group and zeros case in the non-HTAD group.

### 3.6. Reoperations

There were seven secondary procedures (41.2%) in the HTAD group and six (27.3%) in the non-HTAD group (*p* = 0.361). These reoperations are summarized in Table 4.

**Figure 5:**
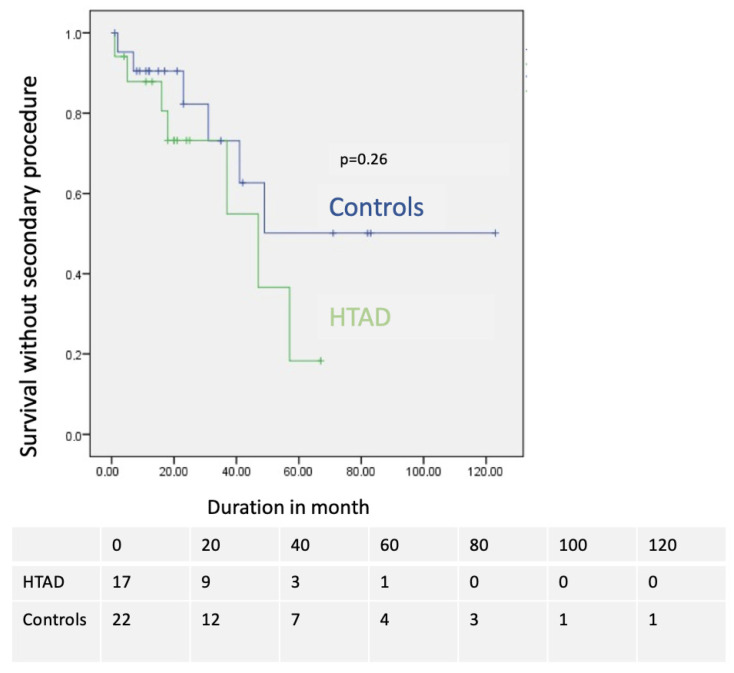
Comparison of secondary procedure-free survival in months in HTAD group (green) and in group 2 (blue).

The mean survival without reoperations was similar in the HTAD group (40.9 ± 6.3 months) and the non-HTAD group (77.2 ± 14.1 months) (*p* = 0.25; Figure 5).

The study of reoperation-free survival in the *FBN1* subgroup (*n* = 10) was performed and showed a median survival at 43.5 ± 6.97 months compared to 77.2 ± 14.1 months in the non-HTAD group (*p* = 0.384).

## 4. Discussion

To our knowledge, this was the first comparative study assessing the outcome of TEVAR in HTAD patients compared to a non-HTAD population.

We observed a more frequent unfavorable anatomical evolution in the HTAD patients with ten cases (58.8%) of significant volume increase compared to five cases (22.7%) in the control patients. In addition, the analysis of diameters at different aortic levels showed a significant increase at the abdominal and celiac levels, suggesting that the unfavorable progression concerned the distal part of the thoracoabdominal aorta below the covered stent graft. In previous studies, Fattori et al. [12] and Faure et al. [13] have shown that TEVAR is associated with a reoperation rate up to 40%, linked to aneurysmal progression of the distal dissected aorta, with an increased risk of distal new entry tears (NETs).

One of the reasons suggested for the increased volume of the distal aorta after TEVAR in HTAD are the NETs, which are more at risk due to the fragility of the tissue. In our study, there were 40% of distal NETs in the HTAD group vs. 20% in the control group. This result contrast with the former occurrence of NETs reported to be up to ten times higher in MFS patients than in non-MFS patients (33% vs. 3%) [14].

Furthermore, as many of the patients were treated with the STABILISE technique (47.1% patients in group one and 31.8% in group two), we performed a subgroup analysis which showed a significantly different increase in the aortic volumes between the two groups of patients with the STABILISE technique, whereas this difference was not observed in the case of simple TEVAR.

These data indicated a poorer outcome of the STABILISE technique in the HTAD patients regarding the increase in the total aortic volumes. In a recent letter [11], Soler et al. reported a risk of aneurysmal evolution after the STABILISE technique in eight patients at fifteen months of follow-up, especially in connective tissue disorders patients.

It has been shown in patients without HTAD that the total aortic volume was significantly higher in patients treated with TEVAR and STABLE compared to those treated with TEVAR alone, and that the increase in the aortic volume was at the expense of the abdominal aorta [15]. It is possible that the stress induced by the STABILISE technique, in the context of HTAD, excessively weakens the aortic wall and was responsible for an unfavorable outcome in the medium and long term.

The permeability of the FL is also a major prognostic factor of AD as shown by Trimarchi et al. [16]. Here, we showed a significant difference in the FL thrombosis between the two groups (52.9% vs. 90.5% in the and non-HTAD groups, respectively, *p* = 0.05). In the literature, in non-HTAD patients, TEVAR for AD is associated with thrombosis of the FL in 90% of patients [17], whereas it was found to be between 70 and 80% in patients with connective tissue disorders [18]. In our study, two patients were treated for malperfusion secondary to residual AD, without closure of the main entry tear, which partly explained this result.

As regards the complications related to the TEVAR technique, we observed no in-hospital mortality (<30 days) in the two groups, which was comparable to the results obtained by Nordon et al. [19]. The occurrence of retrograde AD during endovascular treatment of AD in the HTAD patients was a major concern. In the present study, there were two type A retrograde AD in group one (11.7%). In comparison, Dong et al. [20] had 11 cases of retrograde AD including 3 patients with Marfan syndrome among 443 patients treated with TEVAR from intraoperative to 36 months postoperative. It is difficult to attribute the causality of late retrograde AD to TEVAR or to the natural course of the disease.

The low rate of morbi-mortality associated with the endovascular treatment in the HTAD patients in our study was of major importance. Indeed, conventional surgery of the descending aorta in these HTAD patients is a real challenge and intra-hospital mortality remains high with a rate of mortality at 10% in high-volume centers [21].

There was no significant difference in the long-term reoperation rate between the two groups although it appeared to be higher in the HTAD group (41.2%) than in the control group (27.3%). The rate of reoperation in the control group was relatively higher than that found in the literature (15%), which could be explained by the fact that these patients were young, which is a known risk factor for reoperation [22].

In our study, male gender was significantly associated with an increased risk of progression at 1 year and at the end of follow-up in the HTAD patients. In MFS patients, male gender is also associated with a higher risk for aortic events than females [23].

The impact of the type of endovascular treatment on aortic remodeling should influence the choice of the technique in HTAD patients. In our study, the volumetric analysis has enhanced a differential remodeling after the STABILISE technique in the HTAD patients compared to the non-HTAD patients. By studying the changes in the thoracoabdominal aortic volume below the stent graft between the 1-year and the last available CT scan, we also observed in the HTAD patients an unfavorable evolution of the total aortic volume in the patients treated with the STABILISE technique as compared to TEVAR alone. Faure et al. [10] found stable aortic diameters at the aorto-iliac level in six of the seven patients treated and an iliac aneurysm in one of the patients, but they did not carry out a volume analysis, the follow-up was shorter, and the number of patients was limited, which may explain this underestimation of the aortic remodeling after the STABILISE technique. Thus, this poor result of the STABILISE technique should make us reconsider its use with care in HTAD patients.

## 5. Limitations

This study had limitations related to the retrospective design of this work. Moreover, the absence of a possible comparison with open surgery limited the interpretation of the results. The small sample size and the length of follow-up limited the power of the study. This was due to the rarity (prevalence <1/5000) of Marfan disease, making it difficult to collect a sufficient number of subjects.

## 6. Conclusions

In this study, endovascular treatment in the HTAD patients compared to a control population was associated with a lower rate of complete FL thrombosis and an increased risk of aortic volume progression at 1 year. This difference appeared to be related to the use of the STABILISE technique, which should be considered with caution in HTAD patients. Endovascular treatment with TEVAR alone was associated with acceptable anatomical results and a low risk of perioperative morbidity and mortality in these high-risk surgical patients. A multicenter study with a longer follow-up is planned to confirm these results.

## Figures and Tables

**Table 1:** Demographic data. Comparison of the characteristics of the populations of HTAD patients vs. non-HTAD patients.

Demographic Data	HTAD *n* = 17	Non-HTAD *n* = 22	*p*-Value
Age, mean (SD)	40 (±12)	57 (±8.7)	<0.01
Male sex, *n* (%)	13 (77)	20 (91)	0.374
Hypertension, *n* (%)	10 (58.8)	21 (95.5)	0.013
Smokers, *n* (%)	10 (58.8)	11 (50.0)	0.584
Diabetis mellitus, *n* (%)	1 (5.9)	1 (4.5)	1.000
Dyslipidemia, *n* (%)	3 (17.6)	5 (22.7)	1.000
Coronaropathy, *n* (%)	0 (0.0)	1 (4.5)	1.000
Valvulopathy, *n* (%)	4 (23.5)	1 (4.5)	0.147
LVEF <55%, *n* (%)	2 (11.8)	1 (4.5)	0.570
COPD, *n* (%)	0 (0.0)	2 (9.1)	0.495
Renal failure, *n* (%)	0 (0.0)	1 (4.5)	1.000
Anticoagulants, *n* (%)	5 (29.4)	11 (50.0)	0.195
**Aortic surgery**			
Type A aortic dissection, *n* (%)	8 (47.1)	13 (59.1)	0.053
Valve replacement, *n* (%)	15 (88.2)	4 (18.2)	0.140
Aortic replacement, *n* (%)	2 (11.8)	14 (63.6)	0.570
**Treatment phase**			
Acute and Sub-Acute phase (14–90 days), *n* (%)	9 (52.9)	7 (31.8)	0.332
Chronic phase> 90 days, *n* (%)	8 (47.1)	15 (68.2)	0.053
**Indication**			
Rupture, *n* (%)	0 (0%)	0(0%)	1
Malperfusion syndrome, *n* (%)	5 (29.4%)	4(18.2%)	0.457
Refractory pain, *n* (%)	2 (11.8%)	0 (0%)	0.457
Refractory hypertension, *n* (%)	0 (0%)	3 (13.6%)	0.457
Rapid aortic growth > 5 mm/6 month, *n* (%)	5 (29.4%)	7(31.8%)	0.457
Aneurysmal evolution, *n* (%)	5 (29.4%)	8 (36.4%)	0.457

HTAD: heritable thoracic aortic disease. COPD: chronic obstructive pulmonary disease. LVEF: left ventricular ejection fraction.

**Table 2:** Procedure detail.

	HTAD *n* = 17	Non-HTAD *n* = 22	*p*-Value
Peoximal neck management surgery, *n* (%)	10 (58.8)	16 (72.7)	0.728
IA debranching, *n* (%)	3 (17.6)	12 (54.5)	0.036
LCCA debranching, *n* (%)	4 (23.5)	14 (63.6)	0.027
LSA debranching, *n* (%)	10 (58.8)	16 (72.7)	0.728
3 supra-aortic trunks debranching, *n* (%)	3 (17.6)	11 (50.0)	0.065
**Proximal landing zone (Ishimaru)**			
Z0 *n* (%)	2 (11.8)	11 (50.0)	0.067
Z1 *n* (%)	1 (5.9)	3 (13.6)	1.000
Z2 *n* (%)	7 (41.2)	3 (13.6)	0.022
Z3 *n* (%)	7 (41.1)	5 (22.8)	0.216
Proximal neck length (mm), mean (SD)	14.7 (±14.6)	28.5 (±22.9)	0.067
Proximal neck diameter (mm), mean (SD)	29.1 (±9.0)	32.1 (±7.6)	0.055
STABILISE, *n* (%)	8 (47.1)	7 (31.8)	0.332
Length of cover (mm), mean (SD)	199.4 (±52.8)	194.1 (±50.5)	0.989
Number of entry tears, mean (SD)	5.8 (±3.1)	4.0 (±2.3)	0.052
Diameter of the main entry tears, mean (SD)	13.6(±7.5)	15.1 (±11.4)	0.908
**Location of the main entry tears**			
Segment 2	1 (5.9)	4 (18.2)	0.267
Segment 3	13 (76.5)	10 (45.5)	0.267
Segment 4	2 (11.8)	6 (27.3)	0.267
Segment 5	1 (5.9)	2 (9.1)	0.267

HTAD: heritable thoracic aortic disease. IA: innominate artery. LCCA: left common carotid artery. LSA: left subclavian artery.

**Table 4:** Secondary procedures in group 1 and 2 patients.

HTAD Group	Secondary Procedure	Time to Reintervention (Months)
Patient 1	Hybrid treatment of the throraco abdominal aorta for aneurysmal progression	18
Patient 2	Type A Aortic dissection and distal TEVAR for aortic rupture	57
Patient 3	Type A Aortic dissection and TEVAR for aneurysmal evolution	1
Patient 4	CT embolization, TEVAR, Iliac branch stent graft for type Ib endoleak and aneurysmal progression	47
Patient 5	TEVAR + CT embolisation for aneurysmal progression	37
Patient 6	TEVAR for aneurysmal progression	16
Patient 7	TEVAR for aneurysmal progression	5
**Non HTAD group**	**Secondary procedure**	**Time to reintervention (months)**
Patient 1	Proximal neck embolisation for type Ia endoleak	2
Patient 2	Hybrid treatment of the aortic arch for aneurysmal progression	31
Patient 3	EVAR leg angioplasty for lower limb claudication	41
Patient 4	Intercarotid bypass for cerebral malperfusion	49
Patient 5	Hybrid aortic arch treatment for aorto-bronchial fistula	7
Patient 6	TEVAR for aneurysmal evolution	23

TEVAR: thoracic endovascular aortic repair. EVAR: endovascular abdominal aortic repair. CT: celiac trunk.

## Data Availability

Theh data underlying this article will be shared upon reasonable request to the corresponding author.

## Abbreviations

MFS	marfan syndrome
HTAD	Heritable Thoracic Aortic Disease
TEVAR	thoracic endovascular aortic repair
AD	aortic dissection
TL	True lumen
FL	false lumen
NET	new entry tears
